# Understanding the complexities of Bluetooth for representing real-life social networks: A methodology for inferring and validating Bluetooth-based social network graphs

**DOI:** 10.1007/s00779-020-01435-x

**Published:** 2020-08-13

**Authors:** Bojan Simoski, Michel C.A. Klein, Eric Fernandes de Mello Araújo, Aart T. van Halteren, Thabo J. van Woudenberg, Kirsten E. Bevelander, Moniek Buijzen, Henri Bal

**Affiliations:** 1 Computer Science Department, VU Amsterdam, Amsterdam, the Netherlands; 2 Behavioural Science Institute, Radboud University Nijmegen, Nijmegen, the Netherlands; 3 Radboud Institute for Health Sciences, Primary and Community Care, Radboud University and Medical Centre, Nijmegen, the Netherlands; 4 Erasmus School of Social and Behavioural Sciences, Erasmus University Rotterdam, Rotterdam, the Netherlands

**Keywords:** Ubiquitous computing, Bluetooth, Social networks, Algorithms

## Abstract

Bluetooth (BT) data has been extensively used for recognizing social patterns and inferring social networks, as BT is widely present in everyday technological devices. However, even though collecting BT data is subject to random noise and may result in substantial measurement errors, there is an absence of rigorous procedures for validating the quality of the inferred BT social networks. This paper presents a methodology for inferring and validating BT-based social networks based on parameter optimization algorithm and social network analysis (SNA). The algorithm performs edge inference in a brute-force search over a given BT data set, for deriving optimal BT social networks by validating them with predefined ground truth (GT) networks. The algorithm seeks to optimize a set of parameters, predefined considering some reliability challenges associated to the BT technology itself. The outcomes show that optimizing the parameters can reduce the number of BT data false positives or generate BT networks with the minimum amount of BT data observations. The subsequent SNA shows that the inferred BT social networks are unable to reproduce some network characteristics present in the corresponding GT networks. Finally, the generalizability of the proposed methodology is demonstrated by applying the algorithm on external BT data sets, while obtaining comparable results.

## Introduction

As an integral part of personal communication devices and integrated in many ubiquitous computing systems, Bluetooth (BT) is one of the most prominent technologies for acquiring social proximity traces. The research community has initiated a transition of research methods to reliably represent real-life social connections [9, 43]. Compared with the long-established practices such as deriving social networks based on questionnaires or diaries, ubiquitous computing solutions promise a faster, cheaper, and larger scale data collection process. BT makes an ideal foundation for performing field research experiments with its unobtrusive nature of collecting data.

However, inferring social networks from BT data comes with significant challenges. First, the collected BT data is subject to random noise and may contain substantial measurement errors [15–18]. Often neglected, these errors are propagated to the next phase of a research experiment, which commonly involves analyzing the properties of the BT inferred networks. The absence of ground truth (GT) networks (or any alternative method) for validating the inferred BT networks is another drawback. In many previous research studies, no ground truth information is available for validation, leaving researchers with under-determined problems and random tuning of network model parameters [51, 52]. As shown in Section 2, the majority of previous studies ignore validation procedures of their inferred networks. They assume that the collected BT data *are* the network itself, while in fact, the inferred networks are derived based on the measured interactions, and are strongly dependent on the quality of the collected data. Many network inference methods are relying on parameter thresholds that are hand-tuned or based on domain expert knowledge. As a consequence, the presented inference processes are suitable only for ad hoc, specific network scenarios. There is an evident lack of research contribution for generic inference procedures and rigorous validation methodologies when deriving social networks based on BT (or other ubiquitous) technology. Finally, the full process of network inference (being based on trial-and-error fashion) is unfortunately rarely reported by previous studies. By presenting only the final description of the network, the readers lose valuable information on the whole process of inferring the social networks that can be beneficial for the design of future BT-based data collection systems.

This paper addresses the abovementioned challenges and considers the process of inferring and validating BT-based social networks as a parameter optimization and social network analysis (SNA) problem. It presents an edge inference–based methodology for obtaining BT-based social networks. In this network inference approach, the network nodes are already known while the challenge is to infer edges that reliably represent “real-life” connections. The proposed methodology for optimal social networks inference from noisy BT data consists of two components. First, a Bluetooth Network Validation Algorithm (BVA) delivers the best estimate of the underlying network based on a brute-force search space of a predefined set of input parameters. The final parameter threshold selection is derived by validating all the inferred BT networks with GT networks, rather than hand-tuning or relying on expert knowledge. All the possible inferred BT networks are labeled with particular *accuracy* as a validation measure. The accuracy gives an indication of the success of the edge-inference process, as it measures to what extent (in terms of present/ absent edges) the generated BT networks represent the corresponding GT networks. Second, a SNA compares the optimal BT networks with their GT counterparts to delve into and report the potential structural network differences. A set of well-known network metrics for both global and local network properties were used in order to conduct the SNA.

The three BVA input parameters are defined considering a set of reliability challenges associated to the BT technology itself. The *connection*_*weight* parameter tackles the issue of false positives in the network inferring process [11], as BT-derived proximity is not always an indicator of real social connection [19]. The *window*_*size* parameter tries to establish the minimum number of days of BT observations to produce reliable networks, an essential point for the battery drain issue at used devices. The *connection*_*type* parameter gives an idea of the influence of a particular network design choice (directed or undirected networks) on the inferred network accuracy.

This paper is an extended version of the work published in [44]. The previous work was broadened by including SNA as part of the proposed methodology, and performing generalizability tests using external BT data sets. The methodology was applied to three distinct BT-based data sets. The motivation of adding SNA comes from the fact that BVA *accuracy* as a validation measure has a limited explanatory power of the actual differences among the GT and BT networks. The generalizability of the BVA algorithm was tested on external BT data sets, besides running the algorithm on the school classes data used in [44]. This is an important step for creating *reproducible* methodology that can be used by other researchers working with BT social networks.

To summarize, the main contributions of this paper are:

- Developed a methodology for inferring and validating BT-based social networks via the BVA algorithm and SNA
- Performed throughout quantitative analysis of the network inferring process
- Proved the generalizability of the BVA algorithm by testing the approach on external BT proximity data

The remainder of the paper is organized as follows. The next section presents previous research on BT-based pervasive systems, showing the limitations of using BT data for interpretation of human behavior and the limitations of the BT technology itself. Section 3 introduces the proximity data set used in this experiment. Deriving both the GT and BT networks is explained in Section 4. Section 5 defines the BVA algorithm. The algorithm’s outputs are thoroughly presented in Section 6. The SNA procedure is demonstrated in the subsequent section. The paper ends by discussing the methodological importance and concluding the presented work in Section 9.

## Background

Understanding the limitations of the BT technology for characterizing human behavior is a first step towards developing reliable BT data collection system, and was already investigated by [10, 11, 39]. The main identified limitations are the person-device uncertainty, the granularity (sample period) of the BT traces, and the bias caused by the particularities of the BT technology itself. The person-device uncertainty looks at the ambiguity of the detected interaction, and questions if this interaction indeed appears between humans. In order to reduce this uncertainty, there are several events that should be detected from the collected BT data and properly labeled for the analysis to come. For example, people forget their devices, the device is malfunctioning or people are coincidentally in proximity with others. The granularity and quality of the collected BT traces depends on the type of pervasive devices that are used in the experiment. For instance, smartphones come with a limited set of BT configuration options, mostly in order to save battery. The standard smartphone configurations restrict high BT data collection granularity, which can be critical to detect short events as already experienced by [1, 38, 39]. Finally, the particularities of the BT technology itself posses some limitations. As shown in [10], the rate of false negatives (not detecting a device that is in proximity) increases when more devices are connecting to a wireless medium.

Numerous research have used BT data for recognizing social patterns, inferring social relationships and creating networking structures [1–6]. In addition, the versatility of BT data have been demonstrated by leveraging BT for context-oriented opportunistic networking applications and epidemic modeling [20–22], or even influencing mental health [7]. The inferred BT networks have been applied to many domains for providing improved networking services [2], designing communication overhead algorithms [4], or modeling social distance measures [20]. Several works in the context of inferring social connections from BT data are described below. Dynamic networks were created using longitudinal multi-modal data in [40], where researchers observed cores and social groups among around 1000 Danish students that have participated in the study. They were able to predict social behavior and patterns (ex. social gatherings and meetings) on multiple timescales. In the NSense project [41], the researchers have developed social interaction model for contextualizing nearness with two functions that model both social interaction and propinquity. They have as well relied on multi-modal data (including BT) for conducting a set of experiments, concluding that connected nodes exhibit symmetric patterns of social interaction, and proved that their functions can model the nearness context. Dynamic social networks were mapped based on BT data in [42], where multiple network metrics were used to quantify changes in network topology over time. The researchers discovered correlation between the egocentric network metrics and the scanning rate, therefore confirmed that research outcomes can be strongly dependent on the BT technology limitations. They emphasized that both scanning rate and missing data need to be taken into consideration when deriving BT networks. Using BT signal strength to distinguish between transient and important social interactions was explored in [28], demonstrating that weak links have a lower probability of being observed at later times. In addition, this work showed that removing links with low signal strength influence the network structure.

Evidently, the interplay between BT technology and social networks has motivated a substantial amount of multifaceted research. However, many of the previous studies have one or more important some shortcomings:

- They fail to use a GT network or any other method for validating the inferred BT networks;
- They make scientific conclusions based on the inferred networks without considering the technological constraints of the BT technology (explained at the beginning of this section);
- They fail to report trial-and-error results during the network inference process, and instead present only a final inferred BT network.

Previous research have developed many methodological approaches for inferring social networks from (BT) data. These are found over different application domains and rely on particular knowledge to infer and measure the quality of the inferred networks. The presented research focuses on a specific subset of network inference, where nodes are known and the task is to perform *edge inference*. Edge inference is commonly approached by evaluating models via prediction, or inferring parameters on an assumed parametric model on the data. The goal of predictive methods, used by [8, 26–28], is to model some predictive aspects of the underlying data in order to infer relationships between entities. On the other hand, the parametric models usually rely on a domain knowledge base in order to construct the BT networks. These models make assumptions on the edge distribution, for example by leveraging maximum likelihood estimation [22]. Another category is non-parametric models that use statistical tests to determine edge weights [23–25].

## Data

The data was collected as part of the MyMovez project, by means of a research app shared among pupils in 21 primary and secondary schools in the Netherlands [12]. This project has generated a unique longitudinal large-scale data set (*N* = 953) during a 3-year period. The set contains sociometric data and surveys, physical activity, BT scans, location, photos and chat conversation data, among others. The data is collected via the *Wearable Lab* consisting of smartphone app connected to an activity tracker.

This study exploits the data collected during the first year of the MyMovez project. This data was collected in three data waves: February/March 2016 (Wave 1—W1), April/May 2016 (Wave 2—W2), June/July 2016 (Wave 3—W3). Each wave consists of five consecutive days, out of which three school days and two weekend days (not in this particular order), labeled as D1–D5.

The participants had the MyMovez app installed on dedicated smartphones that were used besides their (possible) private devices during the measurement periods. On the starting day of the experiment, researchers have instructed the children on how to use the handed-in materials, i.e., the smartphone and the activity tracker. Each participant received a Motorola Moto G (gen 2) smartphone and Fitbit Flex (gen 1) activity tracker.

### BT networks data set

The BT data collection logic has been programmatically implemented in the MyMovez app. The app scans and detects other participants’ phones that are in range of approximately 10 m. Total 50 BT scans were performed during each day. A new scan was run every quarter-hour, starting at 07:00 until 19:00. The scan periods were labeled as S0–S49. The time span of each scan period varied between 3 and 5 min, during which nearby devices were able to detect each other. Each BT entry (row) in the database contains the following information: School, Class, Wave, Day, Date, Time, Child_ID, Detected_Child_ID.

Data cleaning procedure was performed in two steps. First, some of the participating classes were removed from further analysis. In total, 953 children and adolescents in 196 unique classes were part of the first year of the MyMovez project. However, as participation in the project was voluntary, participation rates in the classes varied. In some of the classes, almost all pupils were enrolled, but in most of the classes only a few of the pupils were participating. To obtain a reasonable reflection of the social process in the classes, only the classes in which the participation level was higher than 60% of the total number of pupils were included. Missing nomination-based data affects the quality of information obtained for all group members. For example, if 40% of class is participating in a nomination-based study, one also knows just 40% of the relations of the participating peers. As a result, the authenticity of the derived GT social networks is questionable. Although there is no clear-cut threshold value to be recommended, this study has used thresholds based on previous reliability estimation studies [46, 50] of peer-nominated data. They have investigated the effects of different participation rates on the reliability of peer nomination data using statistical measures, which give an estimate of the degree to which nominators agreed upon which nominees best fit a given nomination criterion. In our study, this resulted in 26 school classes that satisfied these participation requirements (60% of class).

**Fig. 1:**
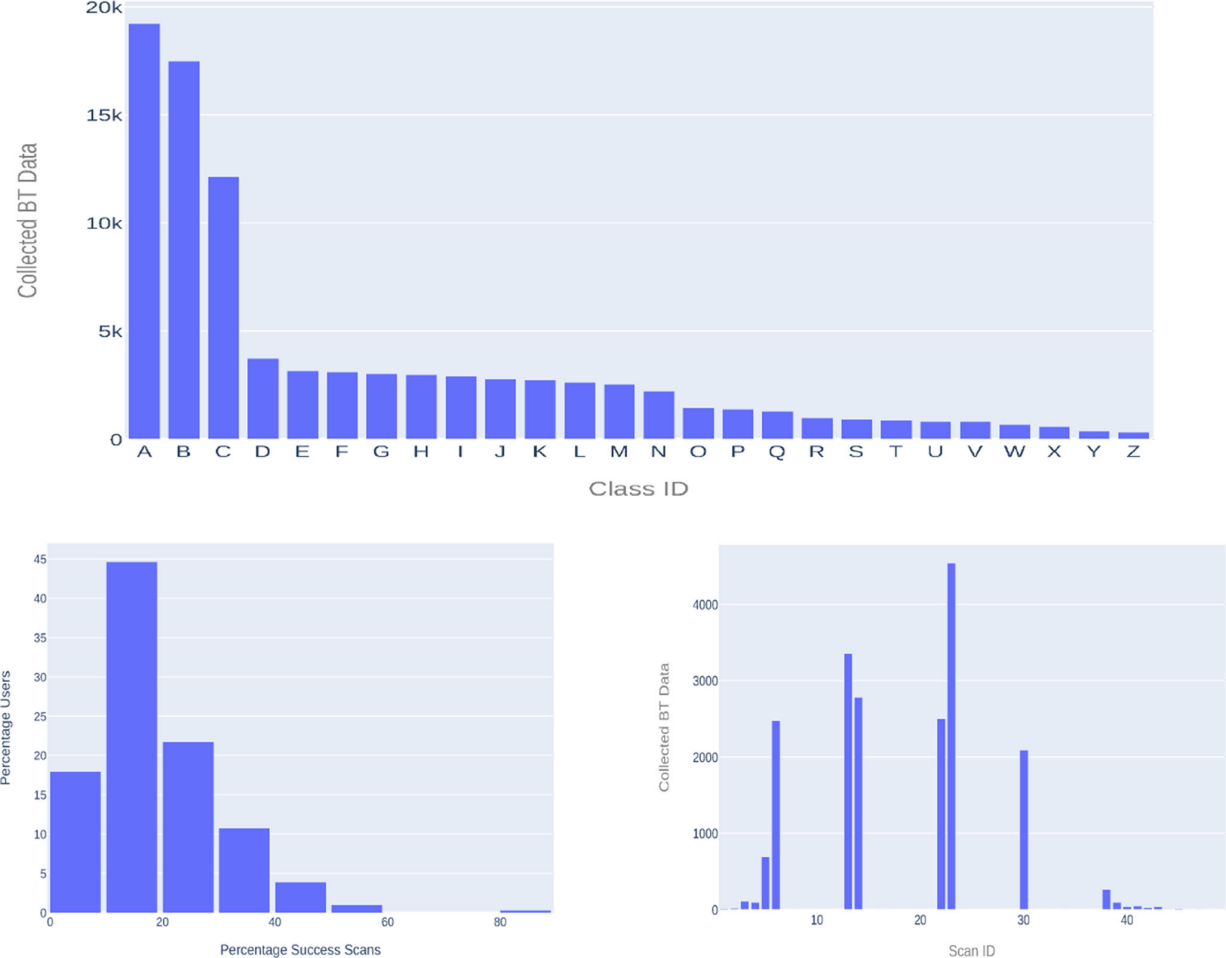
BT data quality statistics. Upper image shows the distribution of the collected BT observations among the 26 school classes labeled from A–Z. The lower left image presents the percentages of successful scans among users. Forty-four percent of participants had collected data in 10–20% of scan periods. The successful scan periods of *ClassA* (aggregated over all waves) are depicted in the lower left figure

In the second step, data quality (availability) analysis was performed on the obtained BT data among the 26 classes, as presented below. There is a significant variation in the data collection quality among these classes, as the number of BT data collected varied between 358 and 19,229 BT observations (all waves combined), as shown in Fig. 1. Classes with IDs A, B, and C had a significantly higher number of BT observations, compared with the rest. Not all 50 scan periods within a day collected BT data (this is valid case for all classes), and the data was not evenly collected by the participants, as shown in the lower left image of Fig. 1. On average, the participants have data in 18% of the predefined scan periods, as the data was primarily collected only before and after school hours, and during class breaks. A further example is shown in Fig. 1 the lower right figure, depicting the scan periods distribution of *ClassA* aggregated over all data waves. BT observations were obtained at only 29 out of 50 possible scan periods per day. The success of a scan period overlaps with particular events happening during that time of the day. For the *ClassA* participants, school day begins at 08:20 in the morning (represented by S5 and S6, at 08:00 and 08:15), first break time is from 10:00 to 10:25 (corresponding with S13 and S14, starting at 10:00 and 10:15). The second break starts at 12:05 until 12:35 (S22 and S23), while the school day ends at 16:05 as represented by S38 in Fig. 1.

The five classes with the highest number of BT observations were selected for the final analysis. The class with ID A is the principal point of the analysis described in the remainder of the paper. Four additional classes were included to test the generalizability of the BVA algorithm, with IDs: B, C (same school with class A) and D, E (belonging to another school).

Table 1 gives some final details on the collected data among the classes selected for the analysis. The participation percentage varies between 62 and 80%. There are sizable differences between the number of BT observations among classes, but also between waves within a class itself.

**Table 1:** Classes data set description

ClassID	Participation (%)	Bluetooth observations (#)			
		Total	W1	W2	W3
A	18/29; 62%	19,229	5560	9558	4111
B	20/30; 67%	17,468	11,117	3050	3319
C	20/29; 69%	12,145	10,347	1484	314
D	20/25; 80%	3734	1794	1822	118
E	19/28; 68%	3165	1822	755	588

### GT networks data set

The participants were asked to complete a set of 16 peer nomination questions at the beginning of each wave. A subset of 6 nomination (sociometric) questions were used in order to derive the ground truth (GT) networks. In these questions, participants were asked to nominate peer(s) from their class that they ask for advice, they consider as leader, they are friends with, they respect, they hang out with, and they want to be like. The exact set of the research validated nomination questions together with their references can be found in Table 2.

**Table 2:** Peer nomination questions

Measure description	Survey question
Advice network [32]	1 item assessing who participants go to for advice
Friends network [34]	1 item assessing who participants are friends with
Leader network [32]	1 item assessing who participants consider as leaders
Respect network [32]	1 item assessing who participants respect
Social facilitation network [33]	1 item assessing who participants hang out with
Want to be network [32]	1 item assessing who participants want to be like

## Building the social networks

### Bluetooth-based social network

**Fig. 2:**
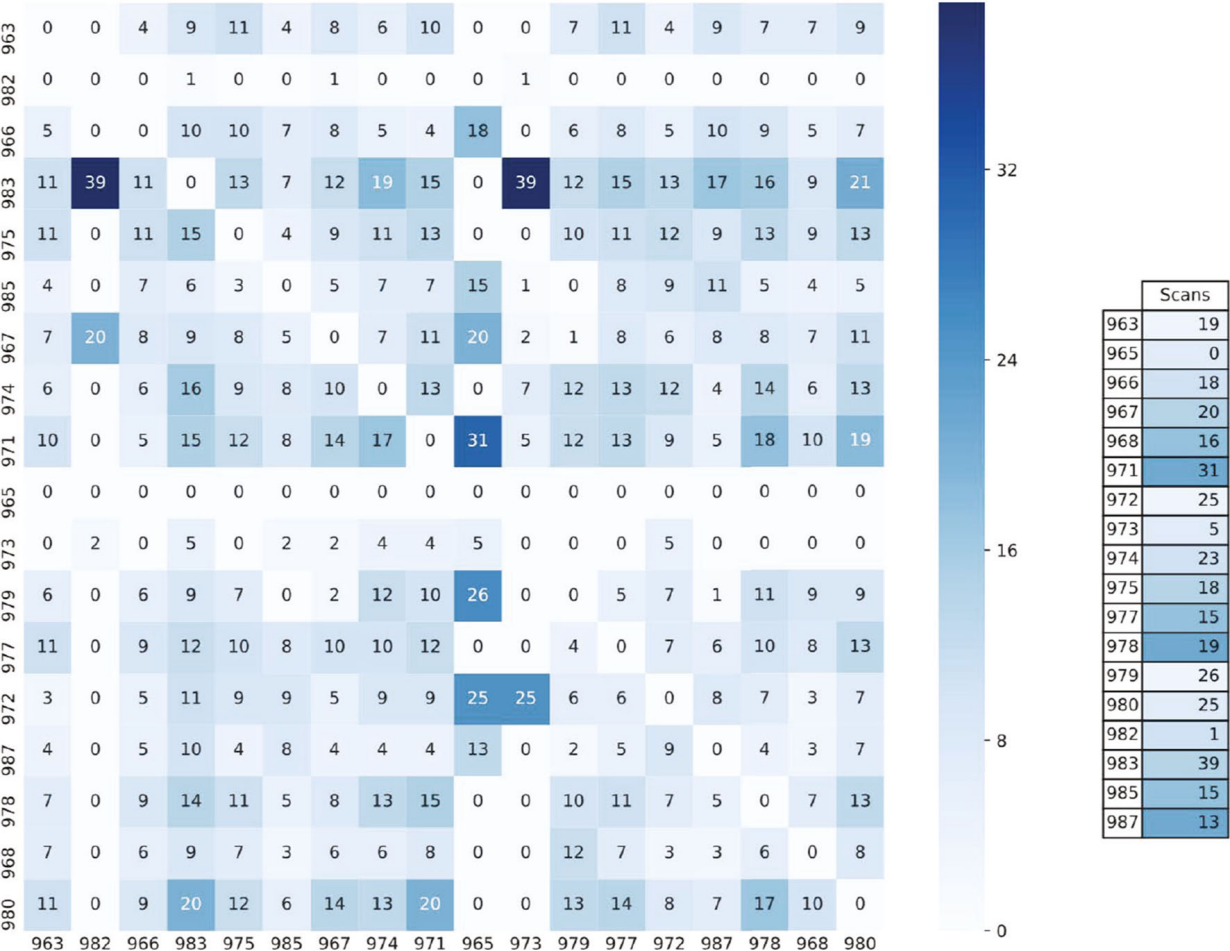
An example connection heat map between all participants of the *ClassA*, based on W2 data. The participants are labeled with three digits starting with 9. Each square of *i* row and *j* column is labeled with *num*_*connections*(*i*,*j*). The table on the left gives information on the number of successful scans per participant

The BT networks are inferred upon the BT data set described in Section 3. The connection weight between two nodes *i* and *j* is defined as:

1 $$  w_{i,j} = \frac{\mathit{num}\_\mathit{connections}(\mathit{i,j}) + \mathit{num}\_\mathit{connections}(\mathit{j,i})+\alpha}{\mathit{num}\_\mathit{scans}(i) + \mathit{num}\_\mathit{scans}(j)+\beta} . $$

where *num*_*connections*(*i*,*j*) is the number of scan periods in which node *i* detected node *j*, and *num*_*scans*(*i*) is the total number of successful scan periods of node *i*. Note that *w*_*i*,*j*_ = *w*_*j*,*i*_ for each connection, and range between [0,1]. Two additional parameters *α* and *β* are included in the weight calculation in order to compensate for the difference in number of scans among the node pairs, as shown in Fig. 2. The problem with the imbalanced number of scans is that naively calculating the weight can assign the same weight for pairs with different scan activity. For example, the same weight *w*= 1 will be calculated for pair (983, 973) and pair (965, 973), even though the *num*_*scans*(983) + *num*_*scans*(973) = 44 while *num*_*scans*(965) + *num*_*scans*(973) = 5. Laplace smoothing [13] was used in order to give the detected connection a certain level of confidentiality and normalize the weight according to the level of nodes’ scan participation. The two parameters *α* and *β* represent the estimation of appropriate weight when no information about the BT observations is available. The parameter values should be fitted to the particular problem scenario. For this weight calculation, *α* = 0 and *β* = 1 as it biases the results towards 0, meaning the weight value is reduced, even more with smaller *num*_*scans* values.

Intuitively, the BT networks can be considered as undirected. The connection between nodes *i* and *j* gives information about their mutual discovery in the physical space, no matter if for some technical reason node *j* cannot detect node *i*. In the undirected BT networks there is a single edge between two nodes. The second network representation (as required by the *connection*_*type* parameter) is the directed BT networks. Here there are two edges between nodes: an edge from *i* to *j* with *w*_*i*,*j*_ and another edge in the opposite direction with *w*_*j*,*i*_, where *w*_*i*,*j*_ = *w*_*j*,*i*_.

### Ground truth social network

Each participant can nominate multiple peers on any of the 6 nomination items presented in Table 2. The label *q*_*n*_*nomination*_*i*,*j*_ represents the nomination of node *i* to node *j* on the particular question *q*_*n*_.

First, the directed GT networks are described. Given two nodes *i* and *j*, the edge *e*_*i*,*j*_ directed from *i* to *j* has weight *w*_*i*,*j*_ based on the number of nominations that node *i* gave to node *j*. Having an edge *e*_*i*,*j*_ does not mean that edge *e*_*j*,*i*_ exists as well. For example, an influential individual might not nominate all the nodes that on the other hand have nominated him/her. The weight of the directed edge from node *i* to node *j* is given by (2).

2 $$  w_{i,j} = \frac{{\sum}_{n=1}^{6} q_{n} nomination_{i,j}}{6}. $$

where ${\sum }_{n=1}^{6} q_{n} nomination_{i,j}$ is the sum of all nominations that node *i* gave to node *j*.

In the second network representation, the GT networks are considered as undirected. In this case, the mutual nominations between node *i* and *j* are summed up and divided by 12 (6 from node *i* and 6 from node *j*) to obtain the strength of their relation. In both network scenarios, the GT network is weighted in range [0,1].

## BVA algorithm

The goal of the BVA algorithm is to find the best solution (the optimal set of input parameters) for deriving an optimal BT network given a certain GT network. Three input parameters are considered during the brute-force search space on the input BT data for inferring BT networks:

1. *connection*_*weight* is used to identify the set of genuine connections in the derived BT networks, rather than “random encounters” connections. Only the edges with *w* >*connection*_*weight* are part of a particular inferred BT network, potentially reducing the noise of random encounters.
2. *window*_*size* gives a time dimension to the inferred BT networks. The window size can be altered on day and scan period(s) during a particular day. This parameter estimates the time and the number of BT observations needed in order to have an reliable representation of GT network.
3. *connection*_*type* considers both directed and undirected social networks. This parameter depicts the consequence of a particular design approach on the accuracy of the inferred BT social networks.

The BVA algorithm was run separately for each of the three data waves (W1, W2, and W3). Each wave represents a distinctive data collection process executed two months apart. A single GT network is generated for each wave and *connection*_*type*, based on the peer nomination questions. On contrary, a large set of BT networks are inferred for each wave and *connection*_*type*, based on the input parameter ranges defined in Table 3. The algorithm infers a new BT network for each combination of the input parameters. The inferring process of a BT network is explained in more detail below. First, BVA considers the *window_size* parameter and subsets the BT data according to the *days* and *scan_periods*. With this, the inferred BT network is given a certain time dimensionality. For example, one BT network is generated based on the BT data collected until Monday afternoon, while another BT network is built upon the whole five days of data collected. In the following step, the connections between peers are created based on the time-subsetted BT data. The connections are given a direction or not, depending on the *connection*_*type* parameter. Finally, the edge weights smaller than the *connection*_*weight* parameter are removed, giving the final inferred BT network.

**Table 3:** Input parameters for the BVA algorithm

Parameter	Ranges
*connection*_*weight*	(0.0–0.6) with stepsize 0.01
*window*_*size*(*day*)	D1, D2, D3, D4, D5
*window*_*size*(*scan*_*period*)	S3, S4, S5, S6, S13, S14, S22, S23
	S30, S38, S39, S40, S41, S42, S43
*connection*_*type*	Directed, undirected

**Fig. 3:**
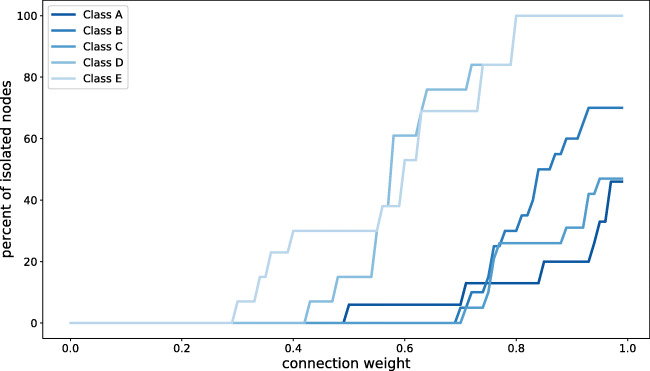
Creation of isolated nodes in networks (expressed as percentage of class size) as a result of increasing the *connection*_*weight*. The BVA is run with *connection*_*weight* between 0 and 1 (step size 0.01) and using all days (*n* = 5) and BT scan periods (*n* = 15) available

The value of *connection*_*weight* was restricted to a maximum of 0.6. From a technical perspective, generating BT networks with higher *connection*_*weight* thresholds result in non-representable real-life scenarios. This is expected from two reasons, the limitations of the BT technology itself and the BT data set incompleteness, which are both explained above. Not all BT scans were successful or collected data about nearby devices, and the used data set have a significant number of missing scans. As example, having a *connection*_*weight* = 0.7 generates a BT network representation where all the connected nodes were scanned and found each other in 70% of the time or *window*_*size*. Having this threshold would remove all the connections between nodes where the *connection*_*weight* < 0.7. The limitations of the data set would therefore result in many edges being dismissed. On the contrary, it is expected that not all school class members have strong mutual connections, but rather the school environment is a mix between weak and strong ties. To further showcase this question, Fig. 3 demonstrates the scenario of running the BVA with *connection*_*weight* ranging between 0 and 1, using all BT scans and days of data, therefore the maximum number of BT observations collected per class. It can be observed that for all five classes, increasing the *connection*_*weight* results in deriving BT networks with a certain amount of isolated nodes (nodes without connections) in the network representations. There is an evident jump in the percent of isolated nodes around the 0.6 point, which served as a motivation to set this threshold. A consequence of dismissing edge connections when *connected*_*weight* is around 0.6 was creation of significant percentage of isolated nodes in the networks, which does not necessarily reflect isolation in the real-life school class, given the above explanation. We consider the isolated nodes as non-realistic, although we realize that there might be specific real-life situations in which social isolation actually exists; however, it is unlikely that this is reflected in isolated nodes generated based on the BT data, as even socially isolated school children will be in the vicinity of other pupils. Therefore, the algorithm generates connection weight values with step size of 0.01 and a maximum of 0.6, resulting in an input set of 60 values.

The *window*_*size* parameter was defined on both day and scan period granularity. All five days were taken into consideration by using the combination formula $\binom {n}{k}=C_{n,k}=\frac {n!}{k!(n-k)!}$ with *n* = 5 and *k* = 1, resulting in the following combinations: (D1), (D1 and D2), (D1 and D2 and D3), etc. In addition, the day granularity was enriched with the scan periods that appeared during a particular combination of day(s). As the BT data is collected unevenly among the scan periods, only the non-trivial scans (at least 30 BT observations per scan) were considered. As a result, 16 scan periods were used with labels as shown in Table 3. The scan periods were combined in a similar manner like days: (S3), (S3 and S4), and (S3, S4, ... SN).

The *connection*_*type* parameter had two variations: undirected and directed networks.

In the second phase of the BVA algorithm, each inferred BT network is validated using the reciprocal GT network, for each wave and *connection*_*type*.

The BVA algorithm’s validation measure is classification *accuracy*. Accuracy is a convenient metric for binary classification problems with (nearly) balanced classes, like the one being solved in this paper. Essentially, BVA checks for edge presence in the inferred networks. The accuracy is defined as:

3 $$  accuracy = \frac{TP + TN}{TP + TN + FP + FN} $$

where *TP* (True Positive) represents the number of edges found in the BT network, also existing in the GT network; *TN* (True Negative) represents the number of edges not found in the BT, also not existing in the GT network; *FP* (False Positive) represents the number of edges found in the BT network, but not existing at the GT network; and *FN* (False Negative) represents the number of edges not found in the BT network, but are found at the GT network. The accuracy value varies in the range of 0 to 1.

## BVA outcomes

The BVA algorithm was run separately per class and data wave. A brute-force search was executed based on the input parameters defined in Table 3. The following analysis gives most details of *ClassA* results, but also presents the outcomes of the other four classes as first step to test the algorithm’s generalizability.

First, the magnitude of the obtained accuracies is discussed. Table 4 gives a detailed overview of the obtained accuracy ranges for the five classes for each wave and *connection*_*type*. The accuracy values vary significantly between the possible BT network representations of particular class and wave. This comes with no surprise given the possible combinations of the input parameters, as 4800 BT network representations were generated for each class, *connection*_*type* and wave. Accuracy variation is therefore expected, as these BT networks are generated with different input parameters (representing different time spans or connection weights), and are all compared with a single GT network representation. For example, the 4800 inferred BT directed networks obtained for ClassA and W1, have an accuracy range between 0.48 and 0.61. This implies an 13% accuracy difference between the least and most accurate inferred BT directed network of ClassA and W1. The highest within-class accuracy range variation is observed at ClassB directed BT networks of W1, with accuracy difference of 22%. On the other hand, the accuracy difference is lowest among the 4800 BT networks obtained for ClassB and ClassC undirected networks (at W2), with only 4%. The detected accuracy variations only confirm that there might be many possible representations of a real-life network, depending on the BT network design decisions.

**Table 4:** Accuracy range of the inferred BT networks

	W1		W2		W3	
Class	DIR	UND	DIR	UND	DIR	UND
A	(.48, .61) #14	(.60, .72) #23	(.49, .58) #16	(.51, .65) #41	(.48, .60) #17	(.63, .74) #32
B	(.37, .59) #16	(.70, .80) #38	(.51, .64) #20	(.74, .78) #13	(.47, .57) #19	(.67, .79) #38
C	(.47, .61) #25	(.55, .75) #70	(.53, .59) #10	(.66, .70) #13	(.41, .49) # 5	(.60, .65) # 7
D	(.48, .60) #11	(.64, .72) #14	(.44, .50) #11	(.57, .63) #19	(.57, .64) # 4	(.73, .76) # 4
E	(.37, .59) #16	(.67, .83) #24	(.33, .47) #12	(.59, .70) #18	(.50, .66) #10	(.69, .85) #19

The observed ranges give the first implication of the BVA generalizability: the accuracies among different classes and waves evidently follow a similar pattern. The undirected BT networks are more alike their GT networks counterpart, compared with the directed BT networks. The ranges of obtained accuracies at undirected BT networks vary from approximately 51 to 85%. On contrary, the directed BT networks have accuracies of 33 to 66%. Table 4 additionally presents the number (#) of distinct accuracy values, i.e., distinct BT networks. This metrics shows that there can be many possible BT network representations inferred from the same BT data source. The number of unique BT networks varies from 4 to 70 among classes, data waves, and *connection*_*type*.

**Fig. 4:**
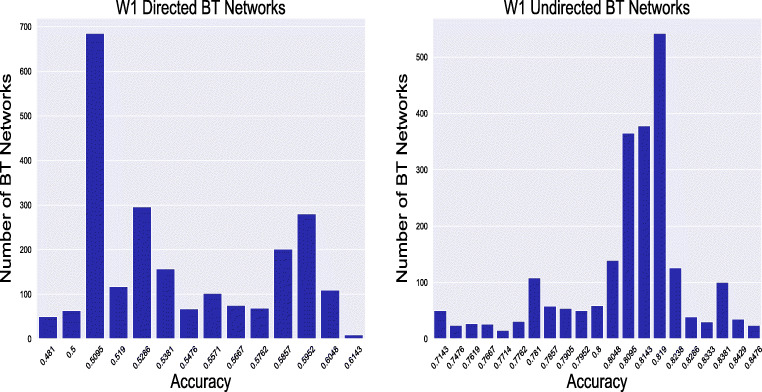
Distinct accuracies and the corresponding number of BT networks, obtained from the BVA algorithm (for *ClassA*). Both W1’ directed and undirected BT networks and their accuracy are displayed

Certain design decisions can therefore lead to selecting an non-optimal BT network. For instance, there are 14 options for choosing a particular directed BT network within *ClassA* W1 results. In this case, there is a 13% potential accuracy loss when the least accurate BT network is selected. This metrics also shows that the number of distinct BT networks is significantly lower than the total number of derived BT networks. The high level of isomorphism among the inferred BT networks is illustrated in Fig. 4. Two networks are isomorphic if they have the same number of nodes connected in the same way. For instance, total 685 derived BT networks of *ClassA* W1 have the same accuracy of 0.50 and 9 BT networks have the identical maximum accuracy of 0.61. The isomorphism among the inferred BT networks is expected given the big granularity of the BVA input parameter set.

The observed isomorphism raises the question of how to select the single optimal BT network among (potentially) more than one optimal BT network? In case the optimal accuracy is equivalent at two or more BT networks, the BVA selects the BT network that was generated with fewer BT observations. In case of another tie at the number of BT observations, the BVA looks at the number of days and scan periods required to derive the networks. In this final case, the optimal BT network is the one being created with fewer number of days and/or scan periods.

The optimal BT networks were selected based on the above described procedure, and are shown in Table 5, along with the parameter values used to obtain them. The *Parameters* column in Table 5 shows the optimal parameter set in the form (W, D, S) representing the *connection*_*weight* value, followed by *window*_*size*’s number of days and number of scan periods, respectively. For example, the optimal *ClassA* W1 BT directed network (with *accuracy*= 0.6143), is derived using BT observations from first two days and 12 scan periods, and including edges with weights bigger than 0.40. The achieved accuracies of the optimal networks are again considerably higher (around 20–25%) at undirected BT networks. The highest optimal accuracy is obtained in the case of *ClassE* W1 undirected BT network with *accuracy* = 0.8545. This BT network therefore represents approximately 85% of the connections in the corresponding *ClassE* W1’s undirected GT network. On contrary, the lowest optimal accuracy is obtained at *ClassC* W2 directed BT network with *accuracy* of 0.4909.

**Table 5:** Optimal parameter sets for achieving highest accuracy per class

	W1			W2			W3		
Class	Parameters	Acc	#BT	Parameters	Acc	#BT	Parameters	Acc	#BT
A DIR	W:0.40;D:2;S:12	.61	5050 (91%)	W:0.54;D:2;S:4	.58	4550 (48%)	W:0.19;D:2;S:8	.60	1972 (48%)
A UND	W:0.40;D:2;S:9	.82	4110 (74%)	W:0.20;D:5;S:3	.65	713 (7%)	W:0.42;D:2;S:7	.74	1375 (33%)
B DIR	W:0.11;D:5;S:9	.59	1734 (16%)	W:0.54;D:5;S:8	.64	2032 (67%)	W:0.18;D:2;S:7	.57	1409 (42%)
B UND	W:0.05;D:2;S:11	.80	9170 (82%)	W:0.35;D:5;S:8	.78	2032 (67%)	W:0.11;D:2;S:0	.79	2897 (87%)
C DIR	W:0.19;D:2;S:14	.61	8319 (80%)	W:0.24;D:5;S:11	.59	1432 (96%)	W:0.33;D:3;S:9	.49	157 (50%)
C UND	W:0.16;D:2;S:10	.85	7115 (69%)	W:0.37;D:2;S:9	.70	615 (41%)	W:0.45;D:3;S:9	.65	157 (50%)
D DIR	W:0.39;D:5;S:7	.60	1412 (79%)	W:0.21;D:2;S:9	.50	1783 (98%)	W:0.59;D:3;S:8	.64	93 (79%)
D UND	W:0.42;D:5;S:8	.78	1773 (99%)	W:0.47;D:2;S:10	.63	1795 (99%)	W:0.59;D:3;S:8	.76	93 (79%)
E DIR	W:0.11;D:5;S:8	.59	1734 (95%)	W:0.12;D:2;S:10	.47	713 (94%)	W:0.24;D:3;S:8	.66	499 (85%)
E UND	W:0.07;D:5;S:10	.83	1882 (100%)	W:0.12;D:2;S:10	.70	713 (94%)	W:0.24;D:3; S:8	.85	499 (85%)

The optimal *connection*_*weight* values vary among waves and network types, and are always higher than 0.0. This confirms that having a certain *connection*_*weight* value removes a level of noisiness in the BT data. The optimal *window*_*size* in order to infer the final BT networks is often less than 5 days. This implies that more days of data collection does not necessarily mean more accurate BT networks. On contrary, two days of BT data are enough to infer 50% of the optimal BT networks (with 15/30 times), followed by five days with 30% of cases (9 times) and three days with 20% of cases (6 times).

Table 5 also shows the number of BT observations (#BT) that were used to obtain the optimal BT networks. Since the number of collected BT observations varies significantly among classes, the ratio of *used* versus *total* BT observations per class is used as an objective statistic. At least 79% of the collected BT data are used to infer the *ClassD* and *ClassE* optimal networks. Unsurprisingly, since these classes have much less data compared with *ClassA*, *ClassB*, and *ClassC*. There is a bigger variety in the percentages of the latter classes, for which the data collection process was more successful. Rarely, more than 80% of the collected BT data was used (at only 5 of 18 cases). Most commonly we observe that about 40-70% of the BT data was capitalized (at 9 cases). In two exceptional scenarios only 7% and 16% of the BT data was enough for inferring the optimal BT networks.

**Fig. 5:**
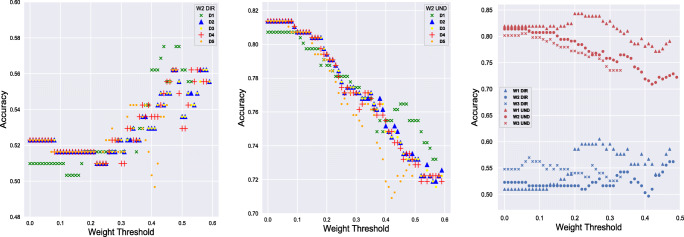
BVA algorithm’s parameter optimization search space in three distinct scenarios of *ClassA*

More detailed perspective of the *ClassA* outcomes is presented next. The visual representation of three particular scenarios of BVA parameter space search are displayed in Fig. 5. The first and second scenario (left and center image) shows the BVA search space for deriving *ClassA* W2 directed and undirected BT networks, respectively. A noteworthy pattern is the reverse nature of obtained accuracies at directed BT networks where the accuracy increases as the *connection*_*weight* increases, as oppose to the undirected BT networks. Different days’ combinations exhibit similar accuracy patterns, however from both figures it is noticeable that having five days of BT data (D5) rarely outperforms the fewer day’s combinations. In contrary, one day of data (D1) is more commonly the best local optimum solution. The rightmost figure displays the obtained accuracies for all waves and *connection*_*type* combinations. Even though the data is collected at different waves (different time periods of the year), there are visible similarities in the BT network accuracies. This is particularly visible by comparing the obtained accuracies at W2 and W3.

Lastly, the BVA optimal scenarios (OS) based on Table 5, are compared with the so-called baseline scenarios (BS). In the BS, the BT networks are inferred without parameter optimization, essentially BVA is not applied: all possible edges (*weight*_*threshold* = 0) and BT observations (*window*_*size* : *day* = 5) are used. Compared with the BS, the OS shows higher accuracy at all data waves. At the W1 directed network the BS achieves an accuracy of 0.50 compared with 0.61 for the OS, in the case of undirected networks the difference is 0.81 for BS and 0.84 for OS. The W2’s directed BS has accuracy of 0.52, as opposed to 0.58 for OS. The W2 undirected BT network is the only case in which the accuracy is not improved, both scenarios have an equivalent accuracy of 0.81. Finally, the W3 directed BS derives accuracy of 0.54, as opposite to 0.60 for OS, the undirected BT networks with minimal improvement (BS: 0.80, OS: 0.81).

## Social network analysis

Accuracy is a good metric for balanced binary classification problems, as in the BVA algorithm, where it signals the presence of a connection in a network. However, *accuracy* doesn’t give a complete picture for the structural differences that might exist among the compared BT and GT networks. For example, even a objectively reliable optimal BT network (e.g., with *accuracy* > 80*%*), might lack a good amount of connections of an important (influential) node. An elaborate analysis on the structural differences between the networks is important when, for instance, researchers want to leverage the network structure to test their scientific hypotheses. To illustrate, one goal of the MyMovez project is to design social network health interventions in the school classes. In this use case, it is important to identify participants that influence the behavior of the peers the most, and select them as influence agents to spread the intervention in the social network. One way to find influential nodes is to observe their degrees of centrality, i.e., look at their connections with others. This is an example when non-detected connections might lead to less optimal set of influence agents being selected, if one is about to rely on the BT networks for testing their hypotheses.

With the aforementioned points considered, this section will delve into the BT and GT social network topology, and report the outcomes of their comparison. Given the magnitude of the presented results in Section 6, the performed social network analysis focused on a subset of the obtained optimal BT networks. The optimal BT networks (*n* = 10) inferred from MyMovez W1 data and their GT counterparts are considered for the presented social network analysis (SNA). The BVA inferred the BT networks with accuracies ranging from 0.59 to 0.61 for the directed, and from 0.80 to 0.85 for the undirected networks (see Table 5).

The BT and GT social networks were quantified using a set of network metrics, namely density, gender-based assortativity, (in-)degree centralization, closeness centralization, and node degree centrality. All these measures were calculated on unweighted BT and GT graphs.

*Density* represents the ratio between the number of edges and the number of all possible edges in a network. More connections among the nodes implies higher network density, with the value of 1 indicating a fully connected network.

*Assortative mixing* in networks gives a measure of the tendency of nodes to be connected to other nodes that are like (or unlike) them in some way [30]. This analysis considers assortative mixing by gender, motivated by previous research that showed that adolescents tend to select friends who are of the same gender [35–37]. The assortativaty coefficient ranges between − 1 and 1 , where 1 indicates perfect assortative mixing (every connection is between nodes of same gender), and − 1 indicates perfect disassortativness (every connection is between nodes of different gender). The coefficient is calculated according to the formulas presented in [30] and depends on the joint probability distribution (mixing matrix) of the specified attribute (gender in this case).

*Centralization* is a network-level measure that gives indication of the standard deviation of individual nodes’ centrality scores. This is different from node centrality measurements, which are based on the individual node in a network. Both (in-)degree and closeness centralization were considered for this SNA. The centralization measures in this analysis are calculated based on the distribution of individual node’s centrality as explained in Freeman’s work on group centrality [31] with ranges between 0 and 1. Centralization value of 1, implies perfectly centralized network, e.g., star network topology. A clique where every node is connected to every other node is clearly not centralized; on the other hand, the star topology, in which only one node *v* is connected to all others and all other vertices are only connected to *v* is a completely centralized graph. High centralization values indicate the presence of pronounced subgroup of nodes with significantly higher individual centrality values compared with the other nodes in the network. Therefore, high centralization can be an indicator of influential (role-model) nodes within the network, and a good measure of the structure of a social network.

*Node degree centrality* is an individual-level measure indicating the number of ties a node has in the network. This measure was used in addition to the centralization metrics from above, in order to get additional information on network structure, this time from individual-level or ego networks perspective.

**Fig. 6:**
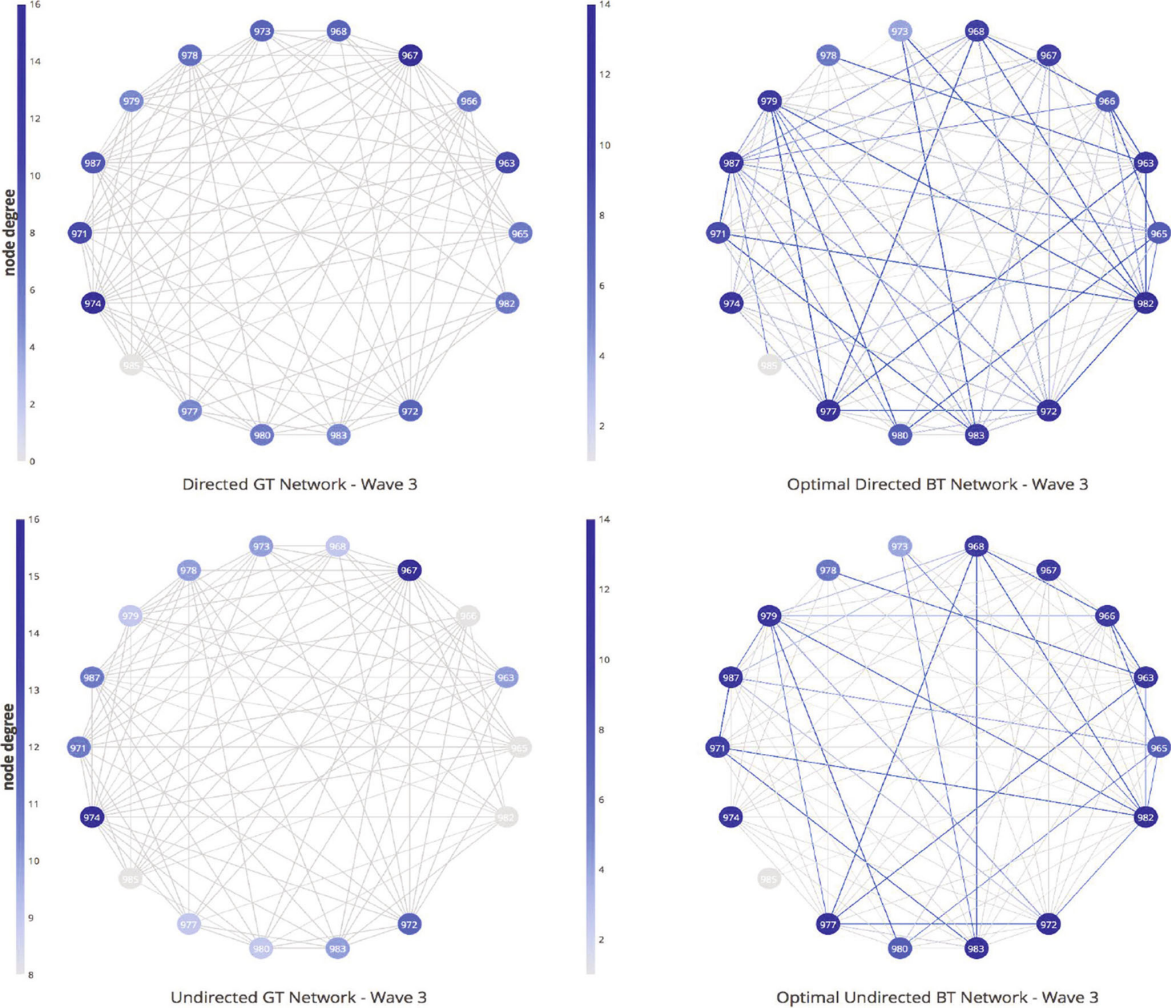
Structural network difference between *ClassA* W1 GT and optimal BT networks. The upper image shows the comparison in case of directed networks, followed by the undirected networks comparison below

The results of the conducted SNA are presented below. Table 6 gives a comparison of the network properties statistics obtained for the optimal BT networks of W1 data and their GT network counterparts. The results are presented in the form GT/BT statistics. All 5 classes and both directed and undirected networks are considered. Comparing BT and GT network *density*, one can conclude that the optimal inferred BT networks tend to overestimate the number of edges present in the GT networks. In most cases, the BT networks tend to be denser compared with their GT counterparts, commonly with differences in range of 10–20%. The density among the BT networks vary between 0.40 and 0.80, and similarly the density among the GT networks vary between 0.45 and 0.85. Certain overestimation of the connections is present at both directed and undirected networks. A visual example of this density difference in Fig. 6 shows the GT and BT networks of *ClassA* (both directed and undirected), where the blue lines in the BT networks (on the right side) illustrate these supplementary edges, that are not found at the GT networks. Table 6 clearly shows a sign of positive *gender-based assortativity* among the GT networks, especially at *ClassA* and *ClassB*. Here, the coefficients are relatively high at both directed networks (0.37 and 0.46) and undirected networks (0.25 and 0.31). On the other hand, the BT networks are not able to capture the assortativity phenomenon, on contrary they frequently show disassortativity (in 7 out of 10 cases). This implies that the nodes might be clustered in a different manner at the GT and BT graphs.

**Table 6:** Four global graph properties (column 3 to 6) are compared among GT and BT networks (W1 data), their values represented as GT / BT in the corresponding columns

Class	Total nodes (% female)	Density	Assortativity gender-based	Centralization (in-)degree	Centralization closeness
A DIR	15 (53%)	0.50/0.63	0.37/0.15	0.23/0.24	0.38/0.15
A UND	15 (53%)	0.68/0.68	0.25/0.08	0.36/0.20	0.50/0.24
B DIR	20 (35%)	0.45/0.67	0.46/0.03	0.25/0.23	0.38/0.15
B UND	20 (35%)	0.60/0.80	0.31/-0.04	0.44/0.16	0.59/0.21
C DIR	19 (26%)	0.56/0.74	0.12/-0.08	0.23/0.15	0.32/0.10
C UND	19 (26%)	0.77/0.77	0.02/-0.07	0.26/0.13	0.38/0.15
D DIR	13 (46%)	0.46/0.48	0.20/-0.08	0.31/0.28	0.41/0.18
D UND	13 (46%)	0.64/0.40	0.12/-0.10	0.42/0.32	0.56/0.39
E DIR	13 (69%)	0.70/0.68	0.17/-0.16	0.15/0.26	0.24/0.18
E UND	13 (69%)	0.85/0.73	0.03/-0.13	0.18/0.22	0.27/0.27

Table 6 gives the evidence that the *centralization* effect of both in-degree and closeness, tends to be higher at GT networks compared with BT networks. In some cases, there is a significant difference, e.g., at *ClassB* undirected GT networks the in-degree and closeness centralization are 0.28 and 0.38 higher, compared with the respective BT networks. It is worth mentioning that the in-degree centralization difference, is more expressed at the undirected networks, while directed networks tend to be more equally centralized. The trends of higher centralization at GT networks (compared with BT networks) are followed by all cases, except at *ClassD* in-degree centralization values. Comparing the centralization values shows that the BT networks could potentially have difficulty capturing subgroups of influential nodes, that can be important for some application scenarios. To further delve into the question of structural difference, the SNA is finalized exploring *node-level centrality* properties. Looking at the individual-level or ego perspective can give details on where those differences occur. For simplicity, this analysis focuses on *ClassA* W1 data, but the results of the other classes are available on request.

**Fig. 7:**
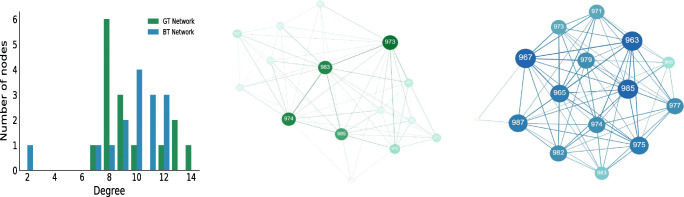
Degree distribution comparison of *ClassA* GT (green) and BT (blue) undirected networks, followed by the topologies of the GT and BT networks. The networks are visualized with different node sizes depending on their degree centrality values, bigger node size implies higher centrality

Figure 7 visualizes the network representations of *ClassA* undirected BT and GT network, and a degree distribution comparison plot, based on the individual-level node centrality. Looking at ego-network perspective can show where exactly the network differences occur. Table 6 shows that there is a big difference at the degree centralization values (GT 0.36 compared with the BT network 0.20), even though they have the same density value of 0.68. The node sizes in the visualized graphs depend on their degree centrality, with higher degree centrality resulting in bigger nodes. Visually, it can be confirmed that the GT network (colored green) is highly centralized with respect to degree, compared with the corresponding BT network (colored blue). There are few nodes, namely 983, 974, and 973 in the GT network, that have much higher centralities compared with the rest of their peers. This is not the case at the BT network, where the centralities are more equally distributed among the members. It is important to mention that different nodes have the highest centralities in their respective networks. At the GT network, nodes 973, 974, and 983 lead with degree centrality of 14, 13, and 12, respectively. The same nodes are among the one with lowest degree centrality if one is to look at the BT network. Here the pairs (node, centrality) look like: (973, 9), (974, 10), and (983, 8). The nodes with the highest degree centrality are 963, 967, and 985 all with centrality of 12. Therefore, it is important to note that even when the BVA derives relatively high accuracy values (in this case 0.82), there can be significant structural differences among the nodes and the derived connections. Lastly, the example of *ClassA* directed networks where the in-degree centralization values of GT and BT are similar (0.23/0.24) reveals a similar conclusion. The most central nodes of the GT network are (983, 10) and (974, 9), while at the BT network the most central is (967, 12). Node 983, the highest centrality valued node at the GT network, is on the lower side in the corresponding BT network with centrality value of 8. Besides degree centrality, eigenvector centrality was additionally considered as part of the SNA. However, it yielded to similar conclusions as the nodes that had the highest eigencentrality were the ones that also have dominated with the highest (in-)degree centrality roles.

## BVA generalizability: application to external data sets

The methodology is designed to be reproducible to other BT data sets scenarios. This section explores the generalizability of the BVA, by running the algorithm on two independent data sets collected in other research experiments.

### SocialBlueConn data set

This data set contains BT proximity data collected by an Android app called SocialBlueConn [29]. The data was obtained from CRAWDAD [45], an open wireless data archive for mobile and pervasive computing. In the SocialBlueConn experiment, the BT observations were gathered in a single data collection wave, from 15 university students during 7 consecutive working days (from January 28, 2014, to February 5, 2014). In addition, the data set includes social profiles, i.e., Facebook friends of the participants that were used in this analysis to derive a GT network.

**Fig. 8:**
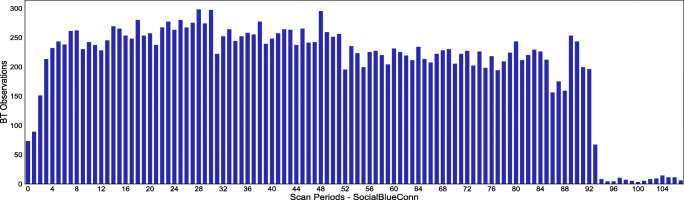
Scan periods distribution—SocialBlueConn data set. The x-axis depicts a particular scan period, while the y-axis gives the corresponding number of collected BT observations. The data is summarized along the 7 collection days

In [29], BT scans are performed on every 3 min, from 12:23 until 17:58, resulting with total 108 scan periods as shown in Fig. 8. The significant drop at the last 20 scans is due to the after-school time. Noticeable, the collected BT observations are more evenly distributed among the scan periods (*M* = 202.96, *SD* = 84.33) compared with the MyMovez data set, as data was being collected throughout the school day. The same applies for the number of BT observations per participant (*M* = 1461.33, *SD* = 217.35). The Facebook friendship data is a 15×15 binary matrix, where 1 indicates a friendship between two nodes.

The BVA algorithm was run on the *SocialBlueConn* data set with some modifications on the input parameter ranges: the *connection*_*weight* was limited to 0.20 and *scan*_*period* in range [1,97]. The *connection*_*weight* range was limited for similar reason as already mentioned in Section 5, however with noticeably lower upper-bound. Higher weight values resulted in non-representative networks rapidly compared with MyMovez data set, possibly due to the more equal distribution of collected BT data among the participants, which produced less variety (and lower values) among the weight values on their connections. The GT networks were generated with connection between two nodes in case there is a Facebook friendship between two participants.

The ranges of obtained accuracies vary from approximately 42 to 76% at directed BT networks, and 71 to 88% at undirected BT networks. This is an indicator of the BVA generalizability, as the accuracy ranges follow similar patterns to the results of MyMovez data set. As a reminder, the accuracies among the MyMovez classes varied between 33 and 66% for directed, and 51 and 85% for undirected networks. The slightly wider accuracy range in the MyMovez data can be expected, given the size of this data set (five classes and three data waves). In total, 37 distinct network representations were generated at both directed and undirected networks, and high isomorphism is once again showed. The potential accuracy loss (as result of a particular network selection) is relatively high, at both directed and undirected BT networks, with 33% and 34% respectively. The optimal networks are derived from the following input parameter combination: *connection*_*weight* = 0.12, *window*_*size*(*day*) = 7, *window*_*size*(*scan*_*period*) = 19, for both the optimal directed and undirected BT network. The exact achieved optimal accuracy were 0.76 and 0.88, for directed and undirected BT networks, respectively. Similar to the reported MyMovez outcomes, having a certain *connection*_*weight* threshold was proven useful. In this case though, the optimal BT networks were generated using the maximal number of days (7 in this data set). Despite the maximum number of days, only 20% of BT observations were used to generate the optimal networks, since only 19 scans were used (out of 108). Comparable with the MyMovez data set, the undirected BT networks showed on average higher accuracy compared with the directed BT networks.

### Copenhagen network study data set

The Copenhagen Network Study (CNS) [48] was conducted among university students; however, unlike the MyMovez and SocialBlueConn data sets, it offers a different magnitude of data, with over 700 university students participating in the experiment. Each participant was given a dedicated smartphone (Google Nexus 4) with an app installed to collect the multi-modal data. The data was collected during a period of four consecutive weeks in 28 days. BT scans were performed every 5 min throughout the whole day, resulting in total 288 scans per day. The GT data is obtained based on the Facebook friendships that were recorded at the end of the experiment.

**Fig. 9:**
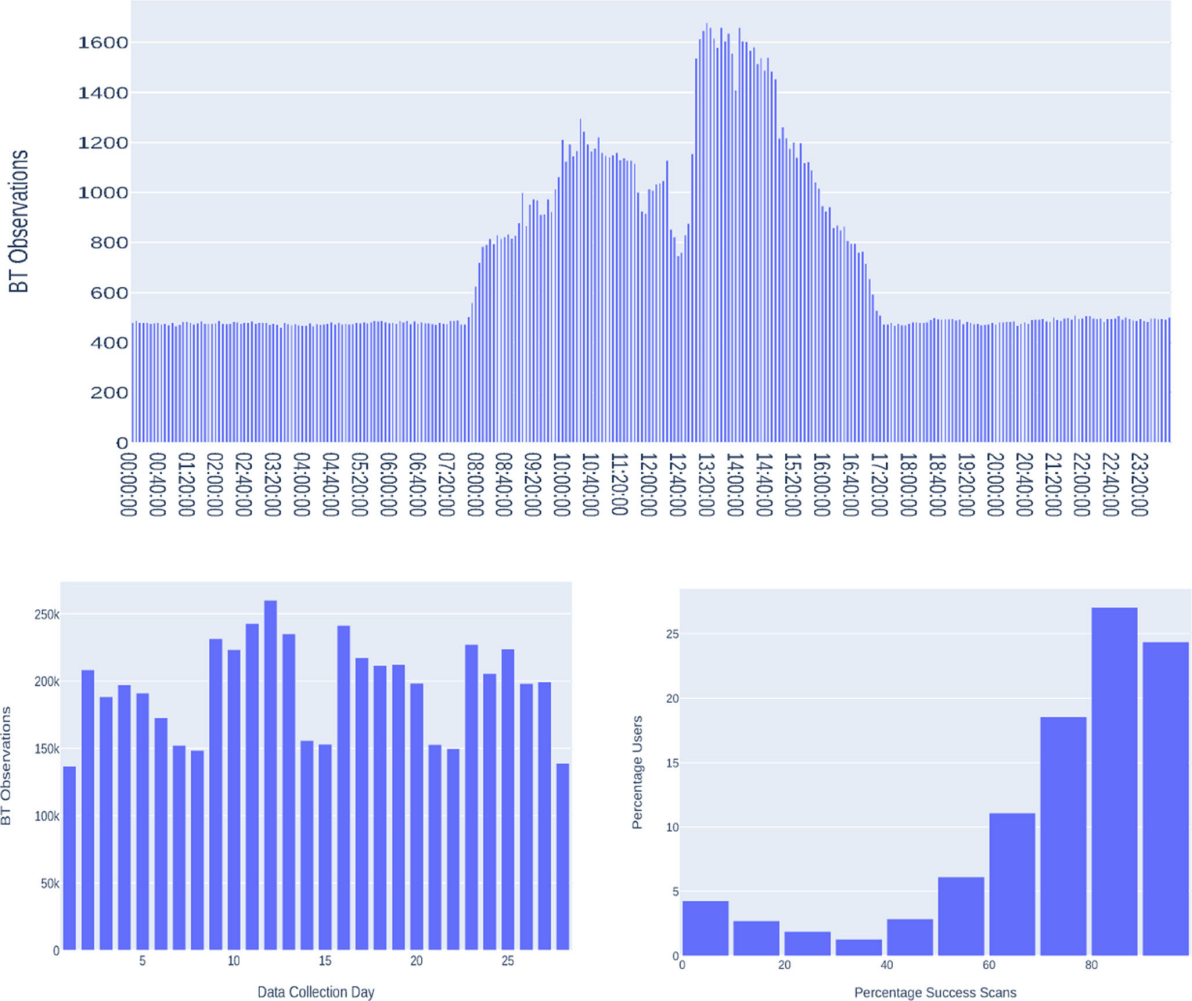
BT data quality statistics of the Copenhagen Network Study. The upper figure visualizes the data collection flow during a particular working day (Monday). On the lower left figure, the distribution of BT observations over the 28 data collection days is displayed. Finally, the lower right figure gives an indication on data collection quality among participants, showing the percentage of successful scan periods

For this analysis, the data collected in the first 7 days (starting from Sunday) was used. In total, 706 users were considered in the analysis, after confirming their presence in both the BT and GT data sets. Figure 9 gives an overview of the conducted BT data quality analysis. The upper image visualizes the BT observations obtained throughout a single working day (in this case Monday). The collected BT data follows an expected pattern of a typical working day: more peer to peer interaction occurring between 08:00 and 17:00, during which university students spend more time together. Similar patterns were observed at the remaining working days, while the weekends are peaking on Saturday evenings. The lower left figure clearly demonstrates that more data is obtained during the working days (Monday–Friday). The data is collected starting on Sunday (the first bar plot) and ends on the 28th day (Saturday—the last bar plot of the figure). Given the size of the experiment, much higher amount of BT data observations are collected as compared with the previous data sets, the average being 195,510 BT observations (*s* = 35441) per day. Finally, the lower right figure gives an overview of the BT data collection quality among the participants. As can be observed, majority of users had successfully scanned over time, with the median being 0.81.

The BVA algorithm was run with *window*_*size*(*day*) in range [*D*1,*D*7], with all possible combinations similarly to the MyMovez data set. All scans during a particular day were taken into consideration, therefore *window*_*size*(*scans*) : [*S*1,*S*288]. The *weight*_*threshold* was limited to 0.5, with a step size of 0.05, giving total 20 combinations. Both directed and undirected social networks were inferred.

The CNS data set has one big difference, when compared with the previous two use cases. It represents highly dynamic network, where nodes are not as interconnected and part of close-knit community like a school class. This becomes obvious when comparing the densities of the obtained GT graphs, between MyMovez and the current data set. For example, the average class density in the MyMovez W1 GT networks was 0.62 (based on Table 6), compared with the GT network density of 0.02 obtained from the CNS data. The low number of reported peer connections is expected given the large-scale university-level participation. However, this indicates that *accuracy* is no longer a representative validation metric, as the CNS use case no longer represents edge inference classification problem with (nearly) *balanced* classes. On contrary, the number of non-edges is significantly larger than the number of present-edges between peers in the network. Therefore, a new validation metric for the BVA algorithm that deals with imbalanced classes scenarios is introduced: the *Matthews Correlation Coefficient (mcc)*. The *mcc* score gives a more reliable statistical rate in case of imbalanced binary classification tasks, compared with using, for example, F1-score as a popular tool in unbalanced classes scenarios [49]. MCC outcome is more realistic as its statistical rate gives a high score only if the prediction obtains good results in all the four confusion matrix categories (TP, FN, TN, and FP), proportional to both sizes of positive and negative elements of the data set. MCC is defined as follows:

4 $$  \mathit{MCC} = \frac{\mathit{TP} * \mathit{TN} - \mathit{FP} * \mathit{FN}}{\sqrt{(\mathit{TP} + \mathit{FP}) * (\mathit{TP} + \mathit{FN}) * (\mathit{TN} + \mathit{FP}) * (\mathit{TN} + \mathit{FN})}} $$

with the score ranging in the interval [− 1,1], where -1 and + 1 indicate the case of perfect misclassification and perfect classification, and the value of 0 is the equivalent of coin tossing (or random) classifier.

The presented results cannot be directly compared with the ones from the previous two use cases, as they are evaluated with different statistics, and as essentially the CNS data set is of different nature. The obtained *mcc* ranges for undirected BT networks vary in between [0.24,0.47], while [0.14,0.36] is the range at the inferred directed BT networks. Similarly to the other scenarios, there are many possible distinct network representations and high isomorphism among the inferred networks. Among the directed BT networks, there are 23 distinct network representations, and 24 for the undirected BT networks. The optimal inferred BT networks are obtained with the following input parameter combinations. For the optimal undirected BT network (with *mcc* = 0.47), the parameter combination *connection*_*weight* = 0.15, *window*_*size*(*day*) = 4, *window*_*size*(*scan*_*period*) = 184, and *connection*_*weight* = 0.5,*window*_*size*(*day*) = 3, *window*_*size*(*scan*_*period*) = 1 for the optimal directed BT network. Once again, the parameter optimization process lead to improvements in the quality of the generated networks. Having a certain threshold on the *connection*_*weight* lead to creating the optimal networks, in addition they are generated with less than 7 days, showing that the BT data collection of 3 and 4 days respectively was enough to obtain optimal inferred networks.

## Discussion

This paper presents a methodology for inferring and validating social networks from noisy BT data. Two main building blocks of the methodology are the BVA algorithm and the SNA. The approach was first tested on a BT data collected among classmates in a school setting. Evidently, the complex BVA search space resulted in many possibilities of representing a social network during the inferring process. There was a high level of isomorphism among the potential BT networks. Therefore, finding the network that best represents the true social connections was based on a set of criteria. In this paper, the optimal inferred networks are those who achieve the highest accuracy (being compared with a GT network) and are obtained from fewer BT observations, and/or fewer days of data collection. The results showed that the accuracies of the optimal BT networks vary largely (between 48 and 85%) among the five MyMovez classes. As anticipated, the accuracy is dependent on the quality of the data collection and the network connection type. Undirected networks were derived with higher accuracy, compared with directed networks.

The *connection*_*weight* was used in order to question the significance of the BT observations as representatives of a real-life social connection. The optimal parameter sets derived from the BVA algorithm (see Table 5) implied that removing a certain extent of edges raises the accuracy of the BT networks. Setting a particular *connection*_*weight* threshold lowers the number of false positives in the networks. Another evident outcome is the large diversity of optimal *connection*_*weight* values. This means that the parameter has to be calculated on a per-case bases, as the level of noisiness of the data is different per scenario. The *window*_*size* parameter revealed that having more BT observations does not necessarily result in more accurate BT networks. On the contrary, the results indicate that two days of BT data collection is frequently a sufficient extent of time to derive BT networks with optimal accuracy. Proper time granularity can be essential for both researchers and participants of an experiment. For the researchers, shorter data collection time makes the system less error prone and eases the data analysis process. The participants’ experience is enhanced, as smartphone battery is saved and they spend less time for the experiment; therefore, the risk of dropout can be significantly reduced. Finally, the effect of a particular *connection*_*type* shows that undirected BT networks are much closer to their GT counterparts, being approximately 20% more accurate compared with the directed BT networks. However, one can argue that these outcomes might be biased by the nature of the social networks, as undirected networks have higher density, therefore reducing the chance for detecting false positives. Therefore, it is important for researchers to consider the potential biases based on their design decisions, and aim for selecting the right network representation for the particular use case of interest.

The second part of the methodology considered SNA for comparing the structural properties of the BT and GT networks. This analysis showed that while accuracy is an appropriate metric, there are considerable structural differences between the BT and GT networks that are hard to be quantified via this classification measure. In conclusion, the inferred BT networks were unable to reproduce some visible GT network characteristics, like gender-based assortativity or degree centralization. Moreover, the subsequent individual-level network analysis showed that different nodes are considered important (influential) at the GT and BT networks. The degree centrality measures ranked different nodes with the highest values at the GT and the BT networks. Nodes that were considered most central at the GT network, were not rarely at the lower side of centrality at the respective BT networks. To conclude, the SNA delivered several meaningful precautions to be considered when using BT networks for testing scientific hypotheses.

To have a reproducible methodology was one of the main goals of this research. Ultimately, the described procedure can be reapplied by developers of similar BT-based data collection systems. In order to test the generalizability of the proposed approach, the BVA was run on two external BT proximity data sets. The first data set, SocialBlueConn, was collected from 15 students’ smartphones in a university setting. Applying the methodology on this data set resulted in obtaining comparable outcomes in terms of accuracy ranges and reliability of the networks. For instance, similarly to the MyMovez data set, there was a high level of isomorphism and potential accuracy loss among the BT networks. Excluding a certain number of potential edges was once again beneficial. Second, the BVA algorithm was run on a data set with slightly different characteristics. The Copenhagen Network Study data set was collected among more than 700 university student participants with a much higher BT data granularity. This network differs from the previous use cases as it does not represent close-knit network, and the edge inference evaluation was updated by including a new metrics, *mcc* for dealing with imbalanced classes. The results once again showed high isomomorphism among the inferred networks, with the highest mcc values obtained being 0.47 for the optimal undirected BT networks, and 0.36 for the optimal directed BT network. More importantly, this use case was useful for expanding the methodology with capabilities to report on imbalanced classification problems.

The presented study also has some limitations. First is the question of missing data. Field experiments are likely to be error-prone and flawless data collection is not expected. One should consider handling missing data, for example, by using machine learning for data imputation, or applying an alternative pervasive technology (in addition to Bluetooth). Furthermore, even though for this research the traditional survey methods are considered as ground truth, they can be erring as well. Human-made mistakes in answering questionnaires can result in erroneous social network graphs. Moreover, the notions of social influence can be larger than only studying friend-based nominations, as considered in this research. For example by social norms: a person can already be influenced by other people that he/she did not nominate, but are in near proximity. An inevitable limitation comes from the nature of the BT technology as already explained in this paper. Using the rich ubiquitous technology ecosystem with the most recent technologies like wearables or BLE-based systems [14] can additionally increase the reliability of inferring network representations from pervasive data. In addition, combining BT data with other data sources like geospacial information or phone contacts can result in more accurate real-life modeling. Another limitation is the lack of data sets that would fit the requirements of the proposed methodology. This is related to several more general observations. First, there is the scarcity of open data in the research community [53, 54]. Second, as already explained in the introduction of this study, previous research rarely went through the process of validating their BT networks; therefore, there is an evident lack of GT network representations. This was confirmed during the process of searching for external data sets for showing the generalizability of the methodology. While CRAWDAD or SNAP [47] are data repositories that offer an impressive number of wireless (and BT) data sets, they were missing GT data counterparts, and consequently were not suitable candidates for validating the BVA algorithm and did not fit the methodology requirements. In the current study, the methodology has been applied to three independent data sets. Although this does not prove generic applicability, it illustrates that the methodology can be applied to different data sets. When more suitable data sets become available, the generalizability of the methodology can be investigated more thoroughly.

To conclude, the obtained results emphasize the need for making the right design decisions and a rigorous methodology *before* deriving BT-based social network graphs. Being able to use reliable pervasive-based technologies like BT for deriving real-life social networks can reduce the overhead of the traditional data collection methods like questionnaires or surveys. Humans are surrounded by technology; therefore, technology-based data collection can seem more natural, compared with the traditional ways of gathering data. It is important to realize that pervasive systems will always result in an *approximation* of real-life social networks, alike the questionnaire-based social network graphs. More evaluative studies on the reliability of using similar technologies can contribute to a better understanding of the proposed systems, and a swift replacement of the traditional methods for deriving real-life social networks.
